# Apology

**Published:** 1856-01

**Authors:** 


*Apology.*—The matter for the December number of the *Journal*, was prepared in good season, and placed in the hands of the printer, with the assurance that the *Journal* should be forthcoming at the proper time. But, very much to our chagrin and annoyance, we could not get a copy from his hands, until the beginning of the second week in January. We, however, commence this number, the first of vol. five, with another printing establishment; and though, owing to the circumstances just mentioned, it is late in its appearance, we trust that we shall have no occasion for further complaints of this kind during the coming year. All letters on business connected with the *Journal*, and communications, should be directed, as heretofore, to Dr. N. S. Davis, Chicago, Ill.

